# Depletion of tryptophanyl-tRNA synthetase and tryptophan accumulation triggers p53-dependent apoptosis

**DOI:** 10.1038/s41420-025-02887-x

**Published:** 2025-12-05

**Authors:** Tayyiba Akbar Ali, Mahmoud Izadi, Raheleh Vazehan, Maryam Al-Mansoob, Ehsan Pourkarimi

**Affiliations:** Division of Genomics and Translational Medicine, College of Health and Life Sciences, Hamad Bin Khalifa University, Qatar Foundation, Doha, Qatar

**Keywords:** Apoptosis, Cancer genetics, Cancer genetics, Disease model

## Abstract

Aminoacyl tRNA synthetases (AaRSs) are enzymes that play a role in maintaining translational fidelity by ensuring the accurate loading of amino acids to their cognate tRNAs. Mutations in the AaRSs are linked to diverse human diseases, including neurological disorders and various types of cancer. Among AaRSs, mutations in *wars-1, a* tryptophanyl tRNA synthetase, have been associated with cancer. Despite the extensive knowledge of WARS-1, there is no comprehensive understanding of its contribution to pathogenesis. In our previous study, we discovered the impact of WARS-1 on genomic integrity. We showed that WARS-1 depletion leads to a significant accumulation of free tryptophan (Trp), resulting in pronounced genomic instability, including the formation of chromatin bridges and micronuclei, and cell cycle arrest. In this study, we demonstrate that *wars-1* knockdown induces apoptosis in the germline of *C. elegans*.

## Introduction

Protein mistranslation occurs once the amino acid sequence of a newly synthesized protein differs from its genetically encoded sequence [1]. Unlike DNA replication, mRNA translation is inherently error-prone, with an estimated error rate of 1 in every 10,000 codons [1, 2]. Mistranslation often arises from errors in mRNA translation due to inaccuracies in tRNA loading. Generally, translational fidelity is ensured by the biochemical process at the core of the translation machinery, leading to the correct loading of each amino acid onto its cognate tRNA. Such precise mRNA decoding is ensured by the family of enzymes known as aminoacyl tRNA synthetases (AaRS) [3]. The primary function of AaRS proteins is to establish a link between the correct amino acids and their genetic codes [3, 4]. All AaRSs possess catalytic and anticodon recognition domains that facilitate the unique aminoacylation processes for their corresponding amino acids. Most AaRSs contain an editing domain, enabling them to proofread and prevent aberrant protein translation that can occur during aminoacylation reactions, providing quality assurance for protein translation [1]. AaRSs are multi-domain proteins and have gained additional domains over the course of evolution, broadening their role in cellular homeostasis [5]. The diverse functions of AaRSs extend beyond mRNA translation, highlighting their importance in various cellular processes and disease pathologies. In addition to their canonical roles, AaRSs participate in various non-catalytic functions, including gene regulation, angiogenesis, autophagy, and innate immunity [6–10]. For example, Alanyl-tRNA synthetase (AARS) in *E. coli* binds to a palindromic region in its gene promoter, resulting in transcriptional suppression and regulation of its expression [10].

Additionally, elevated levels of methionyl-tRNA synthetase (MARS) are reported to increase oxidative stress by promoting homocysteinylation of various proteins, such as superoxide dismutase (SOD1/2), contributing to the pathology of congenital heart defects [11]. Such additionally acquired roles of AaRSs can be mediated by leveraging additional domains of these enzymes or are perhaps associated directly with their active aminoacylation site [12]. Importantly, the noncanonical activities of AaRSs also depend on their cellular localization, alternative splicing, and extracellular secretions [13].

Mutations in various members of the AaRS protein family are increasingly being recognized to be associated with neurodevelopmental disorders [14–18]. More recently, mutation and/or alteration in AaRSs have also been reported to be linked with various types of cancer and cancer progression [19]. Among AaRSs, tryptophanyl tRNA synthetase 1 (WARS1), which is crucial for the attachment of tryptophan (Trp) to its corresponding tRNA, is frequently mutated in various cancers, including lung, breast, and ovarian, as reported in the cBioPortal database (https://www.cbioportal.org/) [9, 20–23]. Mutations in multiple domains of *WARS1* have been identified in other cancer types, including cervical squamous carcinoma, colorectal cancer, and cutaneous melanoma [20, 21]. In gastric cancer, reduced expression of *WARS1* is associated with an increase in lymph node metastases, lymphatic invasion, and poor overall survival [24]. In hormone receptor-positive breast cancer (HR+), elevated *WARS1* expression enhances the tumor sensitivity to docetaxel. In contrast, decreased expression of *WARS1* results in increased cell viability and reduced cell death following docetaxel treatment, making WARS1 a potential prognostic marker and a target for enhancing chemosensitivity [25].

We have previously shown that the knockdown (KD) of *wars-1* in *Caenorhabditis elegans* (*C. elegans*) significantly reduces germ cell proliferation within the mitotic zone, resulting in sterility [17]. This observation aligns with the impact of human *WARS1* loss-of-function mutations, which are associated with microcephaly, a condition that is recognized as a cell cycle disorder [17]. Additionally, we have shown that *wars-1* KD induces cell cycle arrest at the G2/M phase, activates DNA damage checkpoints, and leads to genomic instability. It is now clear that the lack of WARS-1 activity leads to chromatin catastrophes, such as the formation of chromatin bridges and micronuclei in embryos and the pachytene region of *C. elegans* germline [26]. Such drastic alteration in chromatin integrity can potentially result in oncogenesis and cancer progression [27]. Emerging research has shed light on the involvement of AaRS in apoptosis, a process whose dysregulation contributes to human diseases such as cancer and autoimmunity [28–30]. Recent studies of the zebrafish nervous system have demonstrated that highly proliferative cells experience significant cell reduction upon HARS KD. The absence of HARS results in the accumulation of unloaded de-acylated tRNAs, activating general control nonderepressible 2 (GCN2) serine/threonine protein kinase, attenuating translation, and triggering the proteasomal degradation of Cyclin D1 (CCND1). Consequently, proliferating cells arrest at the G1 phase of the cell cycle, ultimately leading to apoptosis [29]. Additionally, isoleucyl-tRNA synthetase (IARS) has been implicated in apoptosis, as melanoma patients exhibit increased *IARS* expression, and downregulation of *IARS* mRNA in melanoma cells results in increased apoptotic death [28].

We previously demonstrated that knocking down *wars-1* combined with supplemental Trp, leads to developmental arrest in *C. elegans*. Moreover, acute Trp exposure activated the DNA damage response, as evidenced by the phosphorylation of CHK-1. Furthermore, metabolite analysis showed that *wars-1* KD results in the accumulation of Trp and its downstream catabolites [26]. Tryptophan is an essential amino acid and is the least used amino acid in nature across all organisms tested so far [26, 31]. Surprisingly, in general, only around 5% of the uptaken Trp is utilized for protein synthesis, while the majority is metabolized to various bioactive molecules that are engaged in physiological processes and maintaining cellular homeostasis [32]. In mammals, Trp is catabolized via two major pathways, i.e., serotonin and kynurenine pathways (KP), with the latter serving as the principal route [33, 34]. Trp is converted into serotonin (5-hydroxytryptamine) by tryptophan hydroxylase (TPH) enzyme, while the KP begins with the breakdown of Trp into kynurenine (Kyn) via tryptophan 2,3-dioxygenase (TDO) and indoleamine 2,3-dioxygenase (IDO1 or IDO2). Kyn serves as a pivotal intermediate of KP and is further converted into kynurenic acid (KA), anthranilic acid (AA), and 3-hydroxykynurenine (HK) via kynurenine aminotransferase (KATs), kynureninase (KYNU), and kynurenine monooxygenase (KMO), respectively. AA and HK are further converted into 3-hydroxy anthranilic acid (HAA) and subsequently converted into quinolinic acid (QA) [33–35]. Another important product of KP is the nicotinamide adenine dinucleotide (NAD^+^), an essential cofactor for cellular homeostasis [36]. Imbalances in Trp metabolism have been associated with various types of cancers and often correlate with poor prognosis. For example, elevated levels of KMO expression in colorectal cancer have been associated with a higher risk of metastasis and reduced patient survival [37, 38]. Increased KP metabolism has been reported in patients with breast cancer, particularly those with soft tissue metastases [35]. Moreover, increased levels of AA and Kyn have been associated with advanced-stage gastric cancer [39]. Kyn has also been shown to promote colorectal cancer cell proliferation [40]. Conversely, Kyn induces cell death in melanoma cells by increasing E-cadherin expression and cleaved caspase-3 activity [41, 42]. Furthermore, reduced levels of Trp can lead to decreased production of serotonin, resulting in depression and cognitive impairment [43]. While studies have explored the effects of altered Trp metabolism, the genotoxic effects of Trp and its catabolites remain poorly understood.

In this study, we aim to investigate the extent of the genomic instability caused by the depletion of WARS-1 and its effect on DNA damage-induced apoptosis. Using high-resolution microscopy, we have shown that *wars-1* KD activates DNA damage-induced apoptosis, mediated by the function of CEP-1, the functional orthologue of the conserved p53 protein in *C. elegans* [44, 45]. Although the *C. elegans* CEP-1 transcription factor did not initially appear to possess high similarity at the protein level, a detailed blast search had identified CEP-1 as the functional orthologue of human p53 [44, 45]. The DNA-binding domain of CEP-1 is highly conserved throughout evolution [44].

The tumor suppressor protein p53 is well known not only for its role as a transcriptional activator of proapoptotic genes, but also for its critical functions in regulating the cell cycle, senescence, and cellular metabolism. A hallmark of cancer is metabolic reprogramming, in which amino acid and glucose uptake are elevated to meet the increased bioenergetic and biosynthetic demands of rapidly proliferating cells. Growing evidence suggests that p53 plays a crucial role in modulating amino acid metabolism, enabling cells to transiently adapt to metabolic stress. In addition to its canonical function in promoting apoptosis in response to genotoxic stress, p53 supports cell survival under nutrient-limiting conditions by regulating the expression of genes involved in glutamine, serine, and arginine metabolism.

In this study, we demonstrate that accumulation of tryptophan and its catabolites following WARS-1 depletion activates the DNA damage response and induces CEP-1/p53-dependent apoptosis. These findings suggest that metabolic imbalance caused by disrupted Trp homeostasis can lead to genotoxic stress, checkpoint activation, and apoptotic cell death, highlighting a potential evolutionarily conserved link between amino acid metabolism and genome surveillance mechanisms. Additionally, we investigated how increased intercellular Trp, resulting from depletion of WARS-1, is linked to cellular viability and genotoxicity-mediated apoptosis. We propose that the effects of WARS-1 are associated with elevated levels of Trp and its catabolites. By silencing key enzymes in the Trp degradation pathways and supplementing with Trp metabolites, we identified specific roles of Trp catabolites, such as kynurenine, in promoting apoptosis.

## Results

### Knocking down *wars-1* induces germline apoptosis

**Fig. 1:**
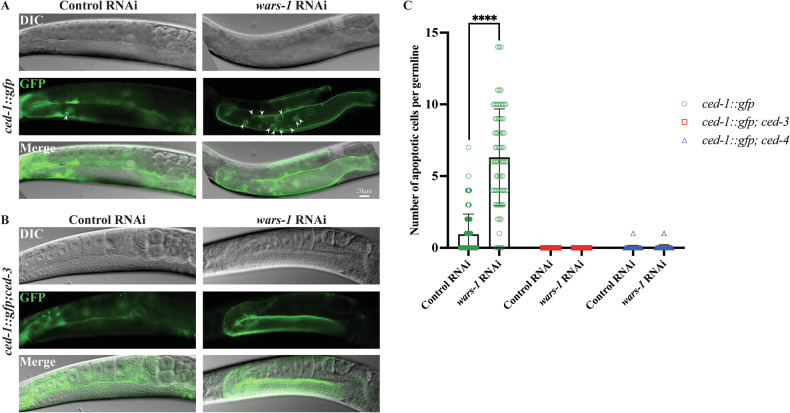
Depletion of WARS-1 leads to germ cell apoptosis. **A** Representative images of the germline of *ced-1::gfp* adult worms on *wars-1* RNAi and the control RNAi. Arrowheads indicate apoptotic cells identified by the apoptotic marker CED-1::GFP. The *wars-1* RNAi treated worm show a significant increase in apoptotic cells compared to the control RNAi in their gonad. **B** Representative images of the germline of *ced-1::gfp;ced-3* adult worms on *wars-1* RNAi and the control RNAi, indicating the absence of apoptotic cells. **C** Quantification of apoptotic cells upon *wars-1* knockdown. The graph depicts the comparison of apoptotic cells induced upon *wars-1* knockdown in *ced-1*::*gfp*, *ced-1::gfp;ced-4*, and *ced-1*::gfp*;ced-3* based on the number of apoptotic cells per gonad. The error bars represent the standard deviation. Statistical significance was determined using a student’s *t*-test (****p < 0.0001), *n* = 60.

To test if the depletion of WARS-1 induces apoptosis, we used RNAi to knock down *wars-1* in a *ced-1::gfp* expressing strain and scored apoptotic cell death in the germline of *C. elegans*. CED-1, a *C. elegans* counterpart of the mammalian Scavenger Receptor from Endothelial Cells (SREC), is a transmembrane protein involved in engulfing dying cells [46]. CED-1 expression and accumulation around the dying cells at the pachytene stage of the *C. elegans* germline is a canonical marker of apoptosis [30, 46]. Treatment of *ced-1::gfp* worms with *wars-1* RNAi resulted in a significant increase in germ cell apoptosis in the pachytene region compared to treatment with the control RNAi (Fig. 1A, C).

During worm development, 131 somatic cells die by apoptosis as part of a normal developmental program. However, apoptosis in worms is not limited to the somatic cells. In an adult hermaphrodite, nearly half of the germ cells undergo a form of natural cell death referred to as physiological apoptosis [47]. Like other organisms, apoptosis in worms is induced by genotoxic insults, which are restricted to the pachytene stage of the *C. elegans* germline. DNA damage-induced and physiological apoptosis share key components of the cell death pathway that are known as the core apoptotic machinery, including the anti-apoptotic CED-9 (the orthologue of the human Bcl-2), the proapoptotic CED-4 (the *C. elegans* counterpart of Apaf1), and the sole *C. elegans* caspase, CED-3 [30, 48, 49]. To determine whether germ cell apoptosis resulting from *wars-1* knockdown depends on the core apoptotic pathway, we assessed the effect of WARS-1 depletion on apoptosis induction using various mutants expressing the engulfment marker, CED-1::GFP. CED-3/caspase is the most downstream component of the core apoptotic machinery and is a cysteine-aspartate protease responsible for executing nearly all apoptotic cell death in *C. elegans* [48]. To test if the observed apoptotic structure upon *wars-1* KD depends on CED-3/caspase activity, we scored apoptotic cells in the *ced-3* (*n2452*) mutant carrying the CED-1::GFP marker. *ced-3* (*n2452*) is a null allele; therefore, apoptosis is abrogated in this background. As expected, no apoptotic cells were detected in the pachytene region of the germline upon *wars-1* RNAi, indicating that the apoptotic structure observed upon WARS-1 depletion depends on caspase activity (Fig. 1B, C). To determine whether *wars-1* RNAi-induced apoptosis requires the core apoptotic pathway, we also measured apoptosis induction upon WARS-1 depletion in *ced-9* (*n1950*) mutant worms. *ced-9* (*n1950*) carries a gain-of-function allele that blocks the apoptosis execution. As expected, the apoptotic phenotype was completely abrogated in the CED-9 gain-of-function background, confirming that the apoptotic phenotype imposed by the lack of WARS-1 depends on the *C. elegans* core apoptotic machinery (Supplementary Fig. S1). To test if such an apoptotic phenotype is restricted to the *C. elegans* germline, we treated the engulfment-defective mutant, *ced-1* (*e1735*), with *wars-1* RNAi. The *ced-1* (*e1735*) mutants are defective in the engulfment and clearance of the apoptotic cells, and the dismantled cells persist, enabling the counting of the persisting dead cells in the somatic tissues, such as in the pharynx [46]. Counting apoptotic cells in the pharyngeal region of *wars-1* RNAi worms revealed no significant difference compared to control RNAi-treated worms (Supplementary Fig. S2).

### Knocking down *wars-1* leads to hyper-accumulation of CED-4/Apaf1 at the nuclear periphery

**Fig. 2:**
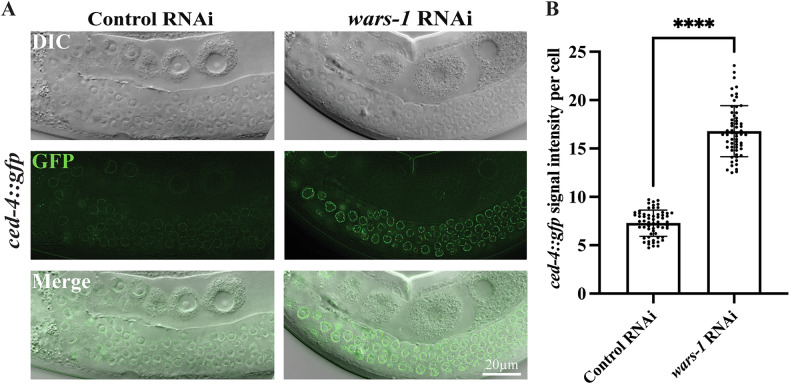
CED-4 hyperaccumulates at the nuclear periphery upon apoptosis induction in *wars-1* KD. **A** Representative images showing the perinuclear hyperaccumulation of CED-4 at the pachytene zone of the germline upon *wars-*1 RNAi and the control RNAi. *ced-4::gfp* hyperaccumulation in the perinuclear region of germ cells upon *wars-1* KD, as compared to control RNAi. **B** The graph depicts the results of *ced-4::gfp* signal intensity per cell upon treatment with *wars-1* RNAi and the control RNAi, measured in cells of the pachytene zone per gonad. Images were taken at 100x magnification, focusing on the pachytene zone. The error bars represent the standard deviation. Statistical significance was determined using a student’s t-test (****p < 0.0001), *n* = 60.

It has been shown that CED-4/Apaf1 hyper-accumulates around the nucleus of pachytene-staged meiotic cells following apoptosis induction [50]. To investigate whether CED-4/Apaf1 plays a role in *wars-1* RNAi-induced apoptosis, we treated the *ced-4::gfp* strain, which expresses exogenous and functional CED-4 protein fused to GFP under the CED-4 promoter and regulating sequences, with *wars-1* RNAi. Treating *C. elegans* with RNAi against *wars-1* increased the accumulation of CED-4/Apaf1 at the nuclear periphery, confirming that CED-4/Apaf1 is involved in apoptosis induction upon *wars-1* RNAi (Fig. 2A, B), which is consistent with our previous findings. Such accumulation of Aapf1-related protein further confirms that *wars-1* KD induces apoptosis in the germline of *C. elegans*. To confirm if the apoptotic phenotype imposed by the lack of WARS-1 is dependent on apoptosome formation, we knocked down *wars-1* in *ced-4* (*n1162*) carrying *ced-1::gfp*. *ced-4* (*n1162*) carries a null mutation that compromises its function. As expected, the apoptotic phenotype diminished with the lack of functional CED-4/Apaf1 (Fig. 1C).

### WARS-1 depletion causes p53 activation

**Fig. 3:**
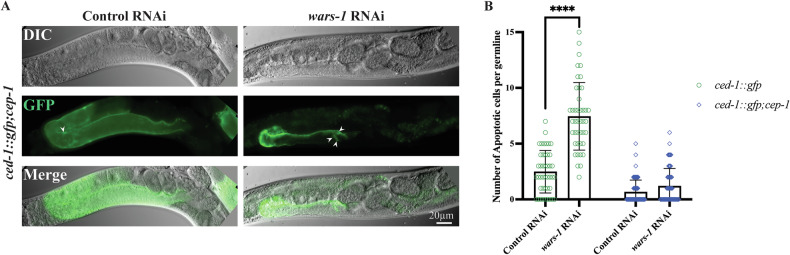
Depletion of WARS-1 leads to DNA damage-dependent apoptosis induced by *cep-1/p53.* **A** Representative images depicting germlines of adult *ced-1::gfp;cep-1* worms, lacking CEP-1/p53 activity. Arrowheads indicate apoptotic cells identified by the apoptotic marker CED-1::GFP. **B** The graph depicts the results of *ced-1*::*gfp* (*n* ≥ 40) and *ced-1::gfp;cep-1* (*n* ≥ 55) upon control and *wars-1* RNAi treatment, based on the number of apoptotic cells per gonad. A significant increase can be seen in *ced-1::gfp* while *ced-1::gfp;cep-1* worms upon *wars-1* RNAi, and the control failed to show apoptosis induction. The error bars represent the standard deviation. Statistical significance was determined using two-way ANOVA (****p < 0.0001). The Brown-Forsythe test was used to assess the equality of variances across groups and resulted in non-significant differences in the standard deviations (SDs) between conditions.

In mammalian cells, genotoxic agents, such as ionizing radiation (IR), ultraviolet (UV) light, and DNA-damaging chemicals such as cisplatin, activate the canonical DNA damage-induced apoptosis mediated by p53. Normally, the expression level of p53 is maintained through various mechanisms, such as ubiquitin/proteasome-dependent protein degradation [51]. Upon DNA damage, the p53 level is elevated, inducing the expression of its target genes to facilitate cell cycle arrest, DNA damage repair, and, in extreme cases, apoptosis [30, 52, 53]. Like in the mammalian system, genotoxic insult induces cell cycle arrest and apoptosis in *C. elegans*, which are mediated by the transcriptional activity of the CEP-1/p53 protein [44]. To test whether *wars-1*-induced apoptosis depends on the *cep-1/p53* activity, we knocked down *wars-1* in the *ced-1::gfp;cep-1 (lg12501)* worm strain. *cep-1* (*lg12501*) is a null allele that cannot induce apoptosis in response to genotoxic insults [44]. Upon *wars-1* KD in *cep-1* (*lg12501*) mutants, the apoptotic phenotype was abrogated, and no significant difference was observed compared to the control RNAi. This provides strong evidence that increased cell death upon *wars-1* RNAi is CEP-1/p53 dependent (Fig. 3). Therefore, our data show that WARS-1 depletion induces apoptosis via *cep-1/p53*, which in turn activates the core apoptotic machinery. Upon activation of the DNA damage checkpoint, the CEP-1/p53 induces the expression of proapoptotic, BH3-only protein EGL-1. To validate the involvement of CEP-1/p53, we measured the expression of its target gene, *egl-1*, using a quantitative real-time PCR (qRT-PCR) in control, *wars-1* KD, and cisplatin-treated wild-types (a positive control). The results demonstrate a three-fold induction of *egl-1* upon *wars-1* KD as compared to the control RNAi. (Supplementary Fig. S3).

### Inhibition of mRNA translation does not promote apoptotic cell death

**Fig. 4:**
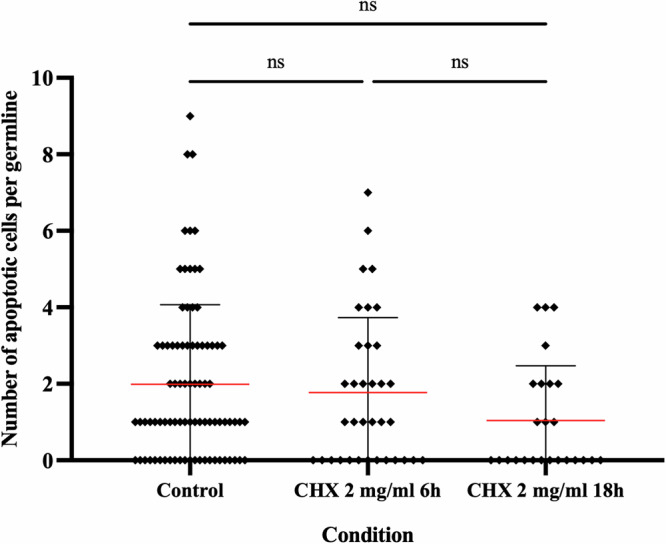
Inhibiting protein translation does not induce apoptosis in *C. elegans* germline. Quantification of germ cell apoptosis of reporter *ced-1::gfp* strain after exposure to 2 mg/ml Cycloheximide (CHX) for either 6 or 18 hours. The red line indicates the mean, and the error bars represent the standard deviation. Statistical significance was determined using two-way ANOVA. (ns: Non-significant), n ≥ 25. The Brown-Forsythe test was used to assess the equality of variances across groups and resulted in non-significant differences in the standard deviations (SDs) between conditions.

Since WARS-1 depletion can potentially impair protein synthesis due to a defect in tRNA aminoacylation, we tested whether the observed germline apoptosis could simply be a consequence of global translation inhibition [54–56]. To address this, we treated *C. elegans* with cycloheximide (CHX), a well-characterized translation inhibitor that blocks ribosomal elongation, to mimic translational arrest independently of WARS-1 depletion. We treated the reporter *ced-1::gfp* strain with 2 mg/ml CHX and measured apoptosis in the germline. Notably, CHX treatment of L4-staged worms for 6 or 18 hours did not result in a significant increase in the number of apoptotic cells compared to non-treated controls (Fig. 4), suggesting that the apoptosis observed upon *wars-1* knockdown is not a general consequence of translation inhibition, but rather reflects a specific effect of WARS-1 loss, likely mediated through imbalanced Trp metabolites. We also have previously shown that blocking mRNA translation using CHX does not activate the canonical DNA checkpoint pathway. These results further support that WARS-1 depletion triggers a distinct cellular response, unrelated to general translation inhibition [26].

### Increased levels of tryptophan promote CEP-1/p53-dependent apoptosis

**Fig. 5:**
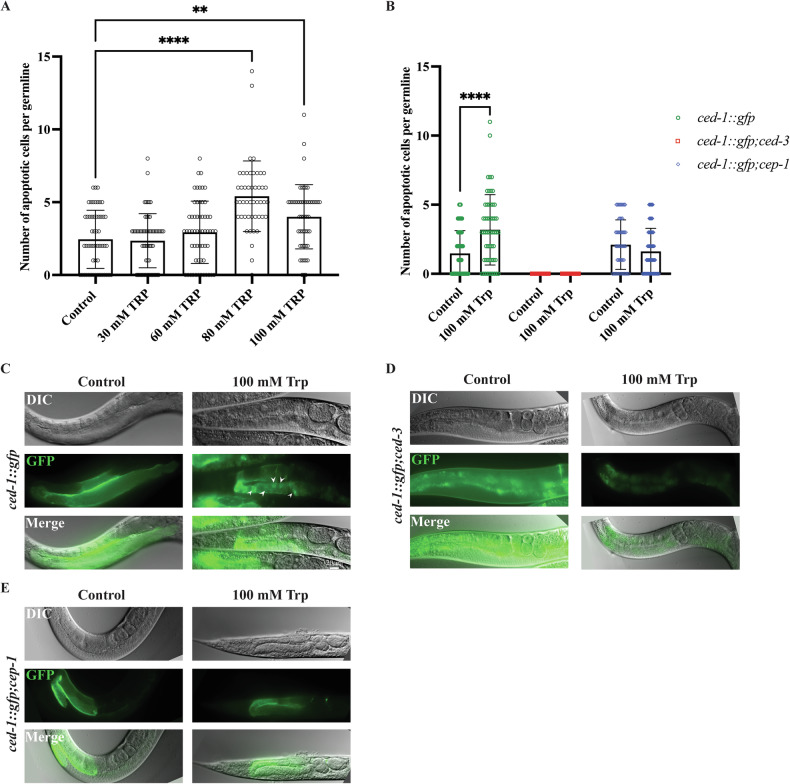
Increased levels of tryptophan induce CEP-1/p53-dependent apoptosis. **A** Number of apoptotic cells per germline upon treating worms with various concentrations of TRP. 80 mM and 100 mM TRP-treated worms showed a significant increase in apoptotic cell number. **B** Quantification of apoptotic cells upon treating worms with 100 mM Trp. The error bars represent the standard deviation. Statistical significance was determined using two-way ANOVA. (****p < 0.0001), *n* ≥ 55. The Brown-Forsythe test was used to assess the equality of variances across groups and resulted in non-significant differences in the standard deviations (SDs) between conditions. **C**–**E** Representative images of the germlines of *ced-1::gfp, ced-1::gfp;ced-3*, and *ced-1::gfp;cep-1* adult worms upon 100 mM Trp treatment and the control. Arrowheads indicate apoptotic cells identified by the apoptotic marker CED-1::GFP.

We previously reported that the *wars-1* KD leads to a four-fold increase in intracellular Trp levels and that exogenous Trp supplementation activates the canonical DNA damage checkpoint mediated by CHK-1 protein [26]. This observation prompted us to investigate whether the apoptosis observed following WARS-1 depletion could be attributed to the cytotoxic effects of elevated intracellular Trp. Therefore, to test this hypothesis, we treated *ced-1::gfp* worms at the L4 stage with various concentrations of exogenous Trp (ranging from 30 mM to 100 mM) until they reached adulthood. Acute treatment of L4-stage worms with 60 mM and 100 mM Trp led to a significant increase in germ cell apoptosis within the pachytene region compared to the untreated control group (Fig. 5A–C). To test whether the observed apoptotic cell death induced by a high dosage of supplemental tryptophan depends on CED-3/caspase activity, we assessed the number of apoptotic cells in *ced-1::gfp;ced-3* (*n2452*) worms. Consistent with our observation, no apoptotic cells were detected in the pachytene region of the germline following 100 mM Trp supplementation. This indicates that the apoptosis induction, triggered by the elevated level of tryptophan, depends on caspase activity (Fig. 5B, D). Previous studies have shown that exposure to a high level of Trp activates the canonical DNA damage checkpoint kinase, CHK-1, suggesting that elevated Trp levels can induce DNA damage [26]. To test if Trp-induced apoptosis depends on p53 activity caused by the upstream DNA damage response pathway, we treated the *ced-1::gfp; cep-1* (*lg12501*) mutant strain with 100 mM Trp. Notably, in *cep-1* (*lg12501*) mutant worms, 100 mM Trp failed to induce apoptosis (Fig. 5B, E). These data strongly indicate that Trp-induced apoptosis requires CEP-1/p53 activity, which is activated by the upstream DNA damage response pathway.

### Disturbance in the Tryptophan degradation pathway results in p53-dependent germ cell apoptosis

**Fig. 6:**
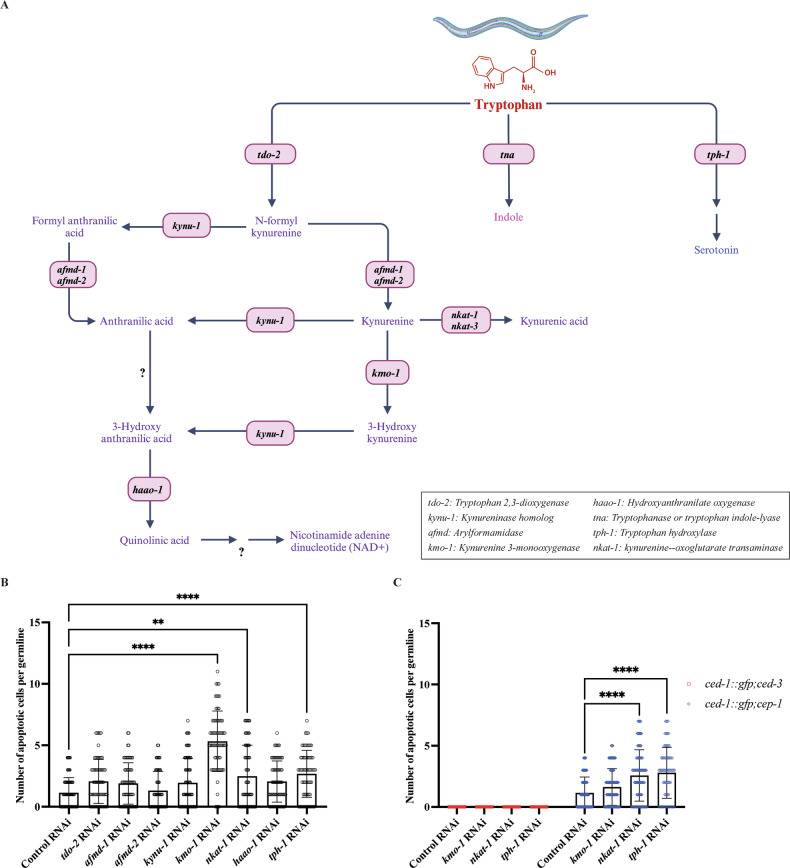
The impact of depleting key enzymes of tryptophan catabolism on apoptosis induction. **A** Schematic diagram of the tryptophan degradation pathway in *C. elegans*. **B** Quantification of apoptotic cells upon treating worms with RNAi targeting various genes of the tryptophan catabolic pathway. Knocking down *kmo-1*, *nkat-1*, and *tph-1* results in a significantly increased number of apoptotic cells compared to the control. **C** Graphical representation of apoptotic cells in *ced-1::gfp;ced-3* and *ced-1::gfp;cep-1* adult worms upon treating with *kmo-1, nkat-1*, and *tph-1* RNAi. *ced-1gfp;cep-1* upon *kmo-1* KD show no difference compared to the control, while *nkat-1* and *tph-1* show a significant increase of apoptotic cells. The error bars represent the standard deviation. Statistical significance was determined using two-way ANOVA. (**p < 0.01; ****p < 0.0001), *n* ≥ 60. The Brown-Forsythe test was used to assess the equality of variances across groups and resulted in non-significant differences in the standard deviations (SDs) between conditions. Tryptophan 2,3-dioxygenase-2 (TDO-2), Kynureninase 1 (KYNU-1), Kynurenine 3-monooxygenase-1 (KMO-1), Arylformamidase 1 (AFMD-1), Arylformamidase 2 (AFMD-2), nematode kynurenine aminotransferase-1 (NKAT-1), 3-hydroxyanthranilic acid oxidase-1 (HAAO-1), and tryptophan hydroxylase-1 (TPH-1).

Across various organisms, tryptophan is predominantly degraded through two major pathways: the serotonin pathway and the kynurenine pathway (KP). Both of which produce a variety of bioactive metabolites. Among these, KP is the primary route for Trp catabolism, accounting for the majority of its degradation [33]. In our previous study, knocking down *wars-1* resulted in a significant increase in the intracellular Trp level, alongside a significant elevation in its downstream catabolites, including HAA and QA [26]. These data strongly suggest that the disruption in the physiological concentration of Trp affects the downstream metabolic flux within the tryptophan degradation pathway, leading to an accumulation of certain Trp catabolites. To test if disturbance in the catabolic Trp pathway, such as the accumulation of specific metabolites or changes in enzymatic reaction dynamics, is associated with DNA damage-induced apoptosis, we systematically investigated the effect of knocking down key enzymes involved in the Trp catabolic pathway. To this end, we used RNAi to knockdown genes coding for key proteins in the Trp catabolic pathway, including tryptophan 2,3-dioxygenase-2 (TDO-2), kynureninase 1 (KYNU-1), kynurenine 3-monooxygenase-1 (KMO-1), arylformamidase 1 and 2 (AFMD-1, AFMD-2), nematode kynurenine aminotransferase-1 (NKAT-1), 3-hydroxy anthranilic acid oxidase-1 (HAAO-1), and tryptophan hydroxylase-1 (TPH-1) in the *ced-1::gfp* strain (Fig. 6A). Interestingly, among all the enzymes involved in Trp degradation, depletion of *kmo-1*, *nkat-1*, and *tph-1* resulted in a significant elevation of the apoptotic germ cells compared to the control (Fig. 6B, Supplementary Fig. S4). To confirm that the apoptotic phenotype is dependent on caspase activity, we measured the number of apoptotic cells in the CED-3/caspase null, *ced-1::gfp; ced-3* (*n2452*) strain, upon knocking down *kmo-1*, *nkat-1*, and *tph-1*. As expected, knocking down these genes in *ced-3* (*n2452*) mutants failed to induce germ cell apoptosis (Fig. 6C, Supplementary Fig. S5A).

Next, to determine if the apoptosis caused by depletion of *kmo-1*, *nkat-1*, and *tph-1* is dependent on the CEP-1/p53 activity, we scored apoptosis cell death in the *cep-1* (*lg12501)* null mutants upon knocking down *kmo-1*, *nkat-1*, and *tph-1*. Interestingly, the apoptotic phenotype of *kmo-1* KD was abrogated in the *cep-1/p53* mutants, compared to control RNAi, indicating that the *kmo-1* RNAi induced p53-dependent apoptosis. In contrast, knocking down *nkat-1* and *tph-1* resulted in an increase in apoptotic death in the *cep-1/p53* null background, suggesting their effect on apoptosis induction is unrelated to the DNA damage and CEP-1/p53 function (Fig. 6C, Supplementary Fig. S5B).

**Fig. 7:**
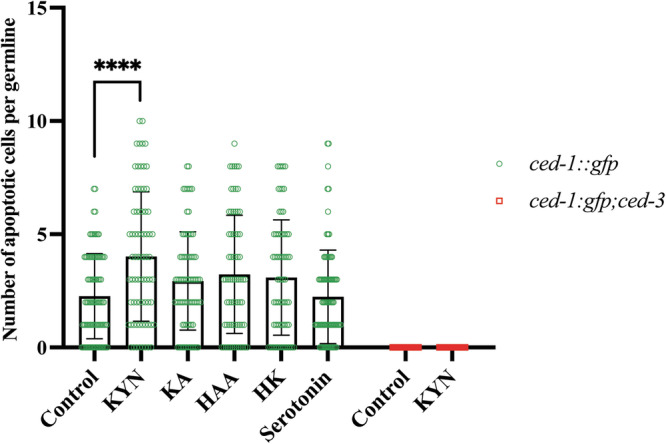
Effect of tryptophan catabolites on apoptosis induction. The number of apoptotic cells in *ced-1::gfp* was quantified upon treatment with various catabolites. Kyn showed a significant increase in apoptotic cells compared to the control in *ced-1::gfp*. The error bars represent the standard deviation. Statistical significance was determined using one-way ANOVA (****p < 0.0001), *n* ≥ 70. The Brown-Forsythe test was used to assess the equality of variances across groups and resulted in non-significant differences in the standard deviations (SDs) between conditions.

TPH-1 converts tryptophan to 5-hydroxytryptophan, the rate-limiting step for serotonin metabolism, while KMO-1 and NKAT-1 are essential in the KP, facilitating the conversion of kynurenine to 3-hydroxykynurenine and Kynurenic acid, respectively. Consequently, knocking down *kmo-1* and *nkat-1* results in intercellular accumulation of kynurenine (Kyn), which may contribute to apoptosis induction. This raises a main question: is an increase in apoptotic cell death observed upon *wars-1* KD driven by the accumulation of Kyn or by its conversion to kynurenic acid (KA)? To address this question, we systematically tested the effect of key metabolites, including kynurenine (Kyn), hydroxyanthranilic acid (HAA), kynurenic acid (KA), hydroxykynurenine (HK), and serotonin, on apoptosis induction. For this aim, we treated L4-staged *ced-1::gfp* worms with the aforementioned metabolites until they reached adulthood. Interestingly, among all the metabolites tested, treating *C. elegans* with supplemental Kyn resulted in a significant induction of germ cell apoptosis, while treatment with KA, HK, HAA, and serotonin did not increase caspase-dependent apoptotic death (Fig. 7, Supplementary Fig. S6). These findings strongly indicate that Kyn has a pronounced effect on inducing apoptosis in the *C. elegans* germline.

## Discussion

In this study, we have explored the effect of knocking down *wars-1* on apoptosis induction. Using various mutants of the apoptotic machinery, we have shown that *wars-1* KD leads to DNA damage-induced apoptosis in the germline of *C. elegans*. This apoptotic phenotype is intrinsically linked to the core apoptotic machinery and is mediated by the activity of the CEP-1/p53 protein. Additionally, we have shown that disturbance in the kynurenine pathway of the tryptophan metabolism leads to p53-dependent apoptotic cell death.

Our previous findings have elucidated the importance of WARS-1 in preserving genomic stability. We demonstrated that *wars-1* KD activates the canonical DNA damage checkpoint, as evidenced by the phosphorylation of CHK-1 at the Ser345 residue [26]. We have also shown that CHK-1 activation following *wars-1* RNAi results in cell cycle arrest at the G2/M phase in the mitotic region of the *C. elegans* germline [17, 26]. Utilizing high-resolution microscopy, we further revealed that the lack of WARS-1 and improper protein translation led to massive genomic catastrophe, which in turn gave rise to various chromatin anomalies, such as the formation of chromatin bridges and micronuclei structures in both mitotic and meiotic cells [26]. Building on our previous observations, this study aims to determine if the intense genomic instability associated with the depletion of WARS-1 and the increase in the intracellular level of Trp and its catabolites leads to apoptotic death. Our findings reveal that knocking down *wars-1* results in apoptotic cell death in the germline, which depends on the activity of CED-3, the sole caspase in *C. elegans*. Notably, knocking down *wars-1* failed to induce apoptotic germ cell death in both *ced-3* and *ced-4*/Apaf1 null mutant backgrounds (Fig. 1). In *C. elegan*s, nearly all apoptotic cell death relies on the core apoptotic machinery comprising the anti-apoptotic CED-9/Bcl-2, CED-4 (*C. elegans* counterpart of Apaf1), and the CED-3/caspase. Importantly, *wars-1* RNAi-induced apoptosis was also dependent on the function of the CED-9/Bcl2 protein, as knocking down *wars-1* did not result in apoptosis in the *ced-9* gain-of-function mutants (Supplementary Fig. S1).

During *C. elegans* development, nearly 131 cells undergo developmental apoptosis as part of a normal worm’s development [57]. Using an engulfment-defective mutant, we found no difference in the number of developmentally regulated apoptotic cells between *wars-1* RNAi-treated worms and the control (Supplementary Fig. S2). This result supports our previous report and indicates that *wars-1* does not influence developmental apoptosis, and its effect is specific to the germline of *C. elegans*. Two distinct death modalities lead to programmed cell death in a worm’s germline: physiological apoptosis and apoptosis induced by DNA damage. Although both pathways depend on the core apoptotic machinery for cell execution, they are activated by distinct upstream signaling [58]. Similar to mammals, approximately 50% of germ cells in *C. elegans* are dismantled by physiological cell death as part of the oogenesis program [47]. Physiological apoptosis is in contrast to DNA damage-induced apoptosis, which is triggered by the conserved DNA checkpoint pathway [59]. Genotoxic insults, such as errors during DNA replication, incorporation of mismatched nucleotides, oxidative stress, ionizing radiation, and exposure to DNA-damaging agents, activate the DNA damage checkpoint pathway. Such activation triggers a transient arrest in cell cycle progression or, under severe conditions, apoptosis [59]. The lack of WARS-1 is reported to be associated with genomic catastrophes such as the formation of micronuclei and chromatin abnormalities, including chromatin bridges [26].

In line with recent findings, we showed that the apoptotic phenotype associated with WARS-1 depletion depends on the activity of CEP-1, the *C. elegans* functional homolog of p53, as *wars-1* RNAi failed to induce apoptosis in *cep-1* null mutants (Fig. 3). While early analyses of the *C. elegans* genome failed to identify a canonical p53 homolog using standard BLAST searches, subsequent work by Schumacher et al. [44] and Derry et al. [45] used sensitive profile-based approaches to reveal that CEP-1 shares a significant relationship to the p53 family, particularly within the DNA-binding domain [44]. These studies established CEP-1 as a divergent, yet functionally conserved, p53-like protein in *C. elegans*. CEP-1/p53 functions as a transcription factor activated through the upstream components of the DNA damage checkpoint, leading to transcriptional induction of its targets, including BH3-only domain proteins such as Puma and Noxa [44, 60, 61].

Similar to the mammalian system, genotoxic insults in *C. elegans* lead to transcriptional induction of the CEP-1/p53 target, *egl-1*, which is the worm counterpart of human Puma [59, 62]. Quantitative real-time PCR analysis demonstrated that knocking down *wars-1* results in a significant transcriptional induction of *egl-1*, similar to the effect of genotoxic agents like cisplatin (Supplementary Fig. S3). These findings are consistent with our previous data and those of others regarding the role of AaRSs on genomic integrity [26]. Recent research on other AaRS proteins has indicated their importance in maintaining genome stability. In general, emerging evidence highlights that defects in loading amino acids to their cognate tRNA can lead to genomic instability and apoptosis, as various mutations in AaRS proteins are reported to activate DNA damage response. A decrease in histidyl-tRNA synthetase (HARS) expression leads to defects in proliferation in the zebrafish nervous system [29]. Importantly, HARS depletion has been shown to increase apoptotic death in the retinal progenitor cells of zebrafish [29]. Additionally, transient expression of mutated valyl-tRNA synthetase (VARS) has been shown to activate p53, which subsequently activates DNA repair pathways [63].

Using targeted metabolomics, we previously demonstrated that loss of WARS-1 results in a ~ 4-fold increase in intracellular tryptophan, accompanied by elevated levels of several downstream Trp catabolites [26]. Notably, this accumulation of Trp and its metabolites was sufficient to activate the canonical DNA damage checkpoint pathway, as evidenced by CHK-1 phosphorylation. This finding is consistent with our observation that exogenous tryptophan supplementation in wild-type *C. elegans* similarly triggers the activation of the DNA damage response, supporting the idea that tryptophan metabolic imbalance results in genotoxic stress [26].

A significant increase in intracellular tryptophan (Trp) is likely due to the accumulation of uncharged, free Trp resulting from impaired aminoacylation activity upon WARS-1 depletion. Since WARS-1 catalyzes the attachment of Trp to its cognate tRNA-Trp, its knockdown is expected to reduce tRNA charging and thereby increase the pool of free, unincorporated intracellular Trp. This elevation in free Trp has been associated with genomic instability and increased levels of Trp-derived catabolites [26]. These findings suggest that excess Trp and its metabolites may contribute to the induction of apoptosis. Consistent with this hypothesis, we observed that supplementing *C. elegans* with increasing concentrations of Trp led to a dose-dependent increase in germline apoptosis, with the most significant effects seen at 80 and 100 mM Trp (Fig. 5A, C).

This Trp-induced apoptosis depended on the activity of CED-3/caspase, as treating *ced-3* mutants with the acute dosage of Trp did not result in increased apoptosis (Fig. 5B, D). Importantly, this apoptosis induction was found to be dependent on CEP-1/p53 function, as treatment with 100 mM Trp failed to induce apoptosis in *cep-1* null mutants, underscoring the genotoxic potential of disrupted Trp metabolism (Fig. 5B, E).

In line with our current findings, we have previously demonstrated that the DNA damage induced by knocking down *wars-1* is specific to this protein. Knockdown of other aminoacyl-tRNA synthetases does not activate the DNA damage checkpoint or induce cell cycle arrest, as evidenced by the lack of CHK-1 and CDK-1 phosphorylation, respectively [26]. Notably, inhibition of protein synthesis did not result in cell cycle arrest or activation of the canonical DNA damage checkpoint [26]. Furthermore, we found that inhibiting protein translation by treating *C. elegans* with cycloheximide failed to induce germline apoptosis (Fig. 4).

While tryptophan is an essential amino acid, it is the least-used amino acid in most organisms [26, 31]. Interestingly, rather than being predominantly utilized for protein translation, most of the absorbed Trp is metabolized into various bioactive components (Fig. 6A), primarily through the kynurenine pathway (KP) and, to a lesser extent, via the serotonin pathway. These bioactive catabolites play crucial roles in various physiological processes governing cellular homeostasis [32]. While the tumor-promoting effect of Trp has been documented in various types of cancer, it is now well-established that physiological shifts in tryptophan flux and its catabolites are implicated in a range of human diseases, including cancer and neurodegenerative disorders [64–68].

Since the majority of Trp is degraded through KP and, to some extent, through the serotonin pathway, we systematically investigated the effect of knocking down the key enzymes in both kynurenine and serotonin pathways. Knocking down key enzymes of Trp degradation revealed that depleting *kmo-1* (kynurenine 3-monooxygenase), *nkat-1* (nematode kynurenine aminotransferase 1), and *tph-1* (tryptophan hydroxylase 1), lead to a pronounced increase in germ cell apoptosis (Fig. 6B, Supplementary Fig. S4). KMO-1, the rate-limiting enzyme in the KP, and NKAT-1 convert Kynurenine to 3-hydroxy kynurenine and kynurenine acid, respectively. Therefore, their depletion results in the accumulation of kynurenine. On the other hand, TPH-1 converts Trp to serotonin, a rate-limiting reaction in serotonin metabolism, most likely resulting in further accumulation of Trp (Fig. 6A). While the apoptosis induced by knocking down *kmo-1*, *nkat-1*, and *tph-1* depends *on* CED-3/caspase, surprisingly, only KMO-1 depletion leads to the p53-dependent apoptotic death (Fig. 6C, Supplementary Fig. S5). Therefore, our data strongly suggest that knocking down *kmo-1*, the most rate-limiting step of KP, leads to the accumulation of kynurenine, leading to CEP-1/p53-dependent cell death. Furthermore, treating *C. elegans* with supplemental kynurenine (Kyn), hydroxy anthranilic acid (HAA), kynurenic acid (KA), hydroxykynurenine (HK), and serotonin demonstrated that only Kyn results in the caspase-dependent cell death, confirming our knockdown genetic data (Fig. 7, Supplementary Fig. S6).

Over the years, numerous studies in human cells have shown that Trp degradation via the KP and the generation of toxic metabolites are associated with cancer progression and tumor formation in the nervous system [66]. It is well documented that Trp catabolites such as 3-hydroxyanthranilic acid (3-HAA), quinolinic acid (QA), and picolinic acid (PA) can inhibit T-cell activity and induce apoptotic death of thymocytes [69]. TDO, the rate-limiting enzyme in the KP, has been shown to promote metastasis in an aryl hydrocarbon receptor (AhR)-dependent manner, and this effect is mediated through Kyn [70, 71]. This finding is in line with the reported elevation of Kyn in cancer patient sera [72]. In lung fibroblasts, Kyn enhances cancer cell growth and migration, whereas treatment with a TDO inhibitor significantly reduces tumor metastasis in mice [73]. In triple-negative breast cancer, suppression of either TDO or AhR was found to decrease proliferation, migration, and invasion [74].

In conclusion, our findings demonstrate that the accumulation of Kyn upon knockdown of *kmo-1* and *nkat-1* leads to significant induction of apoptosis. By systematically testing the effects of key tryptophan catabolites, including Kyn, HK, KA, HAA, and serotonin, on apoptosis in *C. elegans*, we identified Kyn as the principal driver of germ cell apoptosis. Such apoptosis was not observed with other tested metabolites. Therefore, our data strongly suggest that the apoptotic response is specific to Kyn accumulation rather than its downstream metabolites. These findings are highly important and relevant in cancer, where dysregulation of the KP is frequently reported. In general, elevated levels of Kyn have been linked to immune suppression, tumor progression, and resistance to therapy in cancer patients, often through its role in creating an immunosuppressive microenvironment and facilitating tumor immune evasion [38, 75, 76]. Our current result suggests an additional layer of complexity, where excessive Kyn accumulation triggers apoptosis. Tryptophan catabolism varies significantly depending on the environmental and tissue-specific context [77]. Understanding the mechanisms of Kyn-induced apoptosis could reveal therapeutic opportunities, particularly in cancers where KP enzymes such as KMO are upregulated, and targeting Kyn accumulation might selectively induce apoptosis in tumor cells while not affecting normal tissues. However, we do not exclude the possibility that the apoptotic effects observed upon WARS-1 depletion may also involve complex and as yet unidentified functions of WARS-1 that are independent of its canonical aminoacylation activity.

## Materials and methods

### *C. elegance* strains and maintenance

All *C. elegans* strains were maintained at 20 °C on the nematode growth media (NGM) and fed with *E. coli* strain (*OP50*), as described previously [78]. The *C. elegans* strains used in this study are as follows: N2 (Bristol) wild type strain, MD701: *bcIs39 V* [*lim-7p::ced-1::gfp + lin-15*(+)], KX89: *ced-4* (*n1162*) *III*; *bcIs39 V* [*lim-7p::ced-1::gfp + lin-15*(+)], KX84: *ced-3* (*n2452*) *IV; bcIs39 V* [*lim-7p::ced-1::gfp + lin-15*(+)], EPD077: *cep-1(lg12501) I; bcIs39 V* [*lim-7p::ced-1::gfp + lin-15*(+)], CB3203: *ced-1* (*e1735*) *I*, MT4770: *ced-9* (*n1950*) *III*, EPD219: [*ced-4p::ced-4::gfp* (*opls219*)]. In this study, the hermaphrodite worms were used as we focused on the mitosis and meiosis events in the germline of *C. elegans*.

### RNAi construction and knockdown

Construction and RNAi KD of the *wars-1* were carried out as described previously [17, 26]. For all the experiments, the *wars-1* RNAi effect was attenuated by using the control RNAi. Since treating L1-staged worms with 100% *wars-1* RNAi has a severe impact on germline development, various dilutions of *wars-1* RNAi were tested across different developmental stages to preserve germline integrity. Treating the L3 staged worms with 90% of *wars-1* RNAi (v/v) was the most effective condition and was used throughout this study [79]. Genes involved in the Trp metabolic pathway: *tdo-2*, *afmd-1*, *haao-1*, *kmo-1*, *kynu-1*, and *nkat-1*, were KD using RNAi clones from the Ahringer *C. elegans* RNAi library [80], the coding sequence of *tph-1*, and *afmd-2* were cloned to the L4440 RNAi plasmid using molecular cloning and utilizing ApaI (R0114S, NEB) and NotI HF (R3189S, NEB) restriction enzyme [26]. The coding sequence of *tph-1* and *afmd-2* were amplified using *C. elegans* total gDNA and specific primers (Supplementary Table S1). All worms treated with RNAi targeting Trp metabolic pathway genes were treated from the L1 stage, except for *tdo-2*, which was treated at the L3 stage to ensure that the worms could reach adulthood with an intact germline. *C. elegans* strains were fed on HT115(DE3) bacteria carrying the L4440 empty vector (control RNAi) and the corresponding RNAi bacterial strains.

### Tryptophan and other metabolites supplementation

The following metabolites were used in this study: L-tryptophan (ab146400, Abcam), L-kynurenine (K8625, Sigma-Aldrich), 3-hydroxy-dL-kynurenine (H1771, Sigma-Aldrich), kynurenic acid (K3375, Sigma-Aldrich), 3-hydroxy anthranilic acid (148776, Sigma-Aldrich), serotonin hydrochloride (H9523, Sigma-Aldrich). NGM plates were prepared with the following final concentrations of supplements: 100 mM L-Trp, 100 μM 3-hydroxy anthranilic acid (HAA), 50 μM 3-hydroxy-dL-kynurenine (HK), 1 mM L-kynurenine (Kyn), 1 μM serotonin hydrochloride, and 150 μM kynurenic acid (KA). The plates were then seeded with *OP50* bacteria. Synchronized L4 staged worms were grown on these plates until they reached adulthood.

### Cisplatin treatment

Cisplatin treatment was performed as previously described [81]. In brief, N2 worms were grown on the NGM plates until reaching the mid-L4 stage. The L4 stage worms were then transferred to the NGM plates containing cisplatin at a final concentration of 60 μg/mL. After 24 hours, worms were collected from the plates and used for RNA isolation.

### Cycloheximide treatment

Protein synthesis inhibition was performed as previously described [26, 55]. Briefly, the MD701 strain was used to assess the impact of cycloheximide (CHX; ab120093, Abcam) induced blockade of protein translation on apoptosis induction. L4-stage worms were exposed in liquid culture to a final concentration of 2 mg/mL CHX in M9 buffer for either 6 or 18 hours. The number of apoptotic cells in treated and non-treated samples was quantified 24 h after the start of the treatment.

### RNA isolation and quantitative real-time PCR (qRT-PCR)

Total RNA was isolated from nearly 3000 synchronized young adult N2 worms. Animals were collected and washed three times using M9 buffer (3 g KH_2_PO_4_, 6 g Na_2_HPO_4_, 5 g NaCl, 1 ml 1 M MgSO_4_, H_2_O up to 1 liter, Autoclaved for sterilization). Worms were then transferred and lysed in 1 ml TRIzol reagent (15596026, Ambion, Life Technologies) using a bead-beating apparatus (BEAD MILL 4, Fisher Scientific). Total RNA was isolated using TRIzol, following the manufacturer’s recommendation. The purification of isolated total RNA was performed using GeneJET RNA Purification Kit (K0731, Thermo Scientific) following the manufacturer’s protocol. 1 µg of purified RNA was used to synthesize the total cDNA from *C. elegans* using the High-Capacity cDNA Reverse Transcription Kit (4374966, Applied Biosystems). For quantitative real-time PCR (qRT-PCR), a 20 µl reaction volume was prepared using the Power SYBR Green Master Mix (A25742, Applied Biosystems), with each reaction containing 1 µg of cDNA. Gene-specific primers were employed to detect the expression levels of *egl-1* and *γ-tubulin* (Supplementary Table 2).

### Scoring apoptotic cells

Scoring apoptotic cells/corpses was carried out as described previously [82]. To score apoptotic cells, worms were paralyzed using 5 µl of 0.5 M levamisole diluted with M9 in a 1:1 ratio on a 3% agarose pad. Apoptotic cells were counted by observing the CED-1::GFP expression in various mutant strains carrying the *ced-1::gfp* in the background. *ced-1::gfp* strain carries a functional CED-1 protein fused to GFP, expressed in the sheath cells of the *C. elegans* germline, which are responsible for engulfing the apoptotic cells [46]. In strains not carrying the CED-1::GFP, the apoptotic cells were counted using Differential Interference Contrast (DIC) optics. To score developmental apoptosis, highly synchronized L3-staged worms were treated with *wars-1* RNAi on the RNAi plates containing 1 mM IPTG. Once worms reached the adult stage, they were collected from the RNAi plate and bleached to collect the embryos. Embryos were incubated in an M9 buffer to hatch to the L1 stage. The L1 worms were used to score the apoptotic cell death of the pharyngeal region. For 100 mM Trp treatment, the germline was affected such that the pachytene region was reduced in size.

### Microscopy and image analysis

Live imaging and counting apoptotic cells were conducted using a Leica DM6 B 3D-Thunder Imager Fluorescent microscope equipped with a C-mount Leica K5 (14401289) DOC TOP sCMOS Camera. Imaging was performed with HC PL FLUOTAR 40x/0.8 DRY and HC PL APO 100x/1.40 OIL objective lenses, capturing z-stacks of approximately 0.6 μm (40x) and 0.3 μm (100x). All images were digitized in 16-bit format. Microscopic images were acquired using Leica Application Suite X software (LAS X version 3.8.1.26810) and analyzed by ImageJ (https://imagej.net/).

### Randomization and blinding

No randomization or blinding was used in the current study.

### Statistical analysis

GraphPad Prism Version 10.5.0 (673) was used to perform statistical analyses and generate graphs. A student’s *t*-test or one or two-way ANOVA tests were used to calculate the means across different groups, and the resulting *p*-values were calculated. We used the statistical significance of *p < 0.05, **p < 0.01, ***p < 0.001, ****p < 0.0001, and ns: not significant. For all the experiments, at least 3 biological replicates were used to assess the statistical significance unless otherwise noted. When the t-test was performed, an F-test was also used to compare the variances for the groups being compared statistically. When an ordinary one-way ANOVA was used to measure the significant differences between conditions, a Brown-Forsythe test was used to assess the equality of variances across groups. Statistical details of the experiments can be found in the results and discussion section and in the figure legends.

## Supplementary information

Supplementary Fig. S1.

Supplementary Fig. S2.

Supplementary Fig. S3.

Supplementary Fig. S4.

Supplementary Fig. S5.

Supplementary Fig. S6.

Supplementary Table S1.

Supplementary Table S2.

## Data Availability

All data generated and analyzed during the current study are available from the corresponding author upon request.
