# Supplementary material for: Depletion of tryptophanyl-tRNA synthetase and tryptophan accumulation triggers p53-dependent apoptosis
Source: Cell Death Discov. 2025 Dec 5;12:34. doi: 10.1038/s41420-025-02887-x (PMC12824228; doi:10.1038/s41420-025-02887-x)
Supplement: Supplementary file 1 — Supplementary Fig. S1. [file 41420_2025_2887_MOESM1_ESM.pdf]

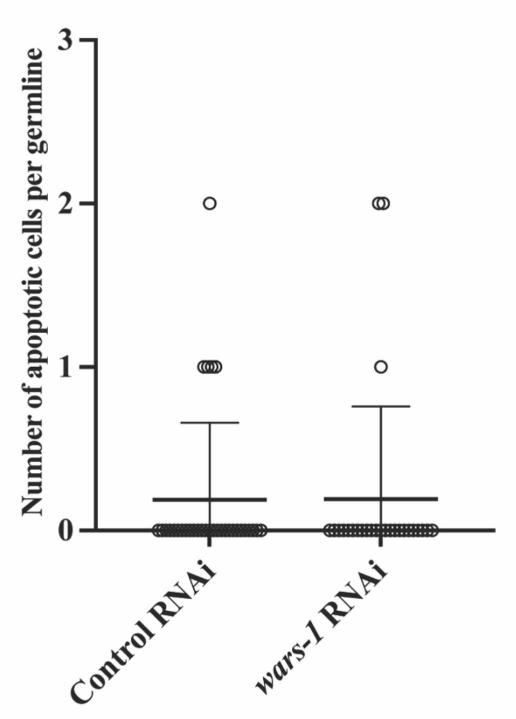

Supplementary Figure S1. Quantification of apoptotic cells in the *ced-9(n1950)* gain-of-function mutant. The apoptotic phenotype observed upon *wars-1* RNAi was completely abrogated in the *ced-9(n1950)* background, confirming that WARS-1 depletion–induced apoptosis depends on the *C. elegans* core apoptotic machinery.
