# Supplementary material for: Depletion of tryptophanyl-tRNA synthetase and tryptophan accumulation triggers p53-dependent apoptosis
Source: Cell Death Discov. 2025 Dec 5;12:34. doi: 10.1038/s41420-025-02887-x (PMC12824228; doi:10.1038/s41420-025-02887-x)
Supplement: Supplementary file 2 — Supplementary Fig. S2. [file 41420_2025_2887_MOESM2_ESM.pdf]

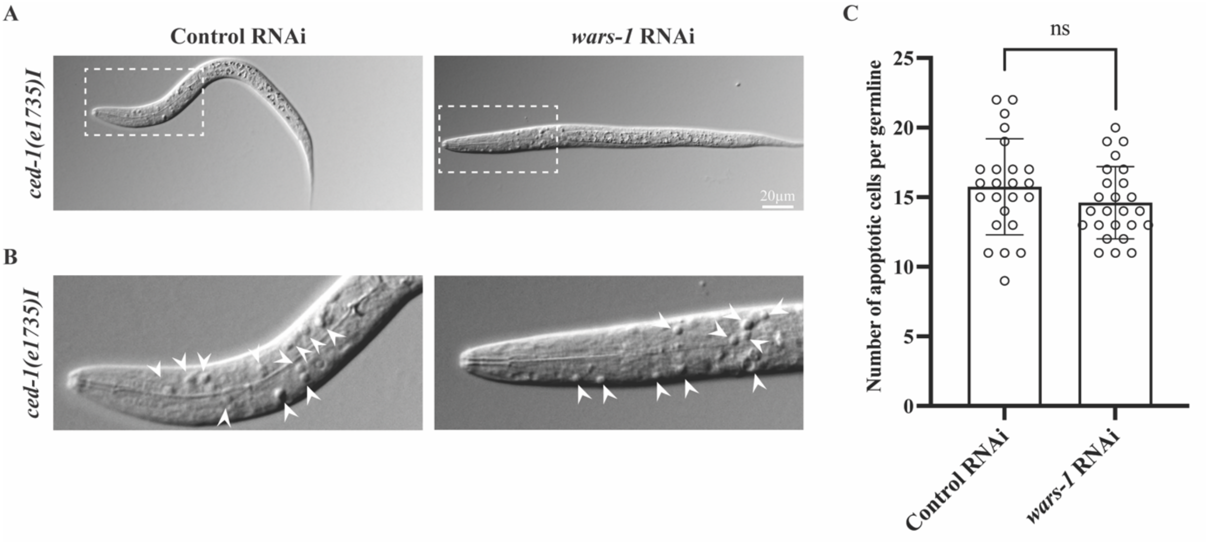

Supplementary Figure S2. Quantification of apoptotic cells in the engulfment-defective mutant *ced-1(e1735)*. Apoptotic cells were counted in the pharyngeal region of *wars-1* RNAi-treated worms and compared to control RNAi-treated worms. No significant difference in the number of apoptotic cells was observed upon *wars-1* depletion. **A.** Representative L1-stage worms treated with either *wars-1* or control RNAi. **B.** Enlarged view of the pharyngeal region indicated by the dashed rectangle in panel A. Arrowheads indicate apoptotic cells. **C.** Quantification of apoptotic cell numbers in the pharynx showing no significant difference between *wars-1* RNAi and control RNAi conditions.
