# Supplementary material for: Depletion of tryptophanyl-tRNA synthetase and tryptophan accumulation triggers p53-dependent apoptosis
Source: Cell Death Discov. 2025 Dec 5;12:34. doi: 10.1038/s41420-025-02887-x (PMC12824228; doi:10.1038/s41420-025-02887-x)
Supplement: Supplementary file 3 — Supplementary Fig. S3. [file 41420_2025_2887_MOESM3_ESM.pdf]

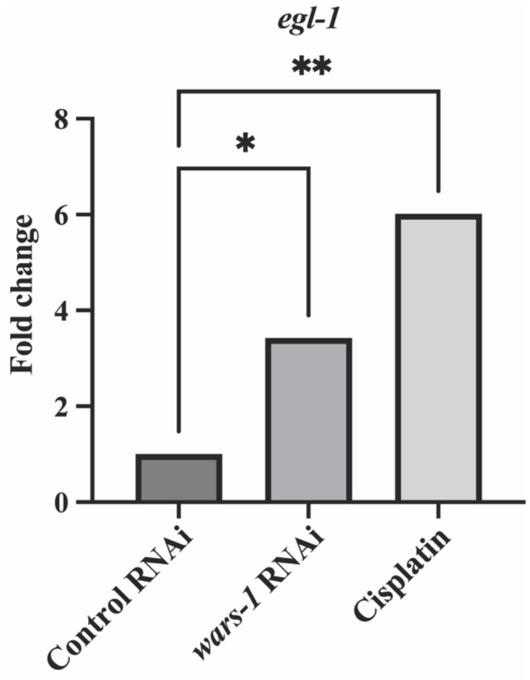

Supplementary Figure S3. Quantitative real-time PCR (qRT-PCR) analysis of *egl-1* expression in wild-type worms treated with control RNAi, *wars-1* RNAi, or cisplatin (positive control). *wars-1* knockdown resulted in an approximately three-fold increase in *egl-1* transcript levels compared to the control, indicating activation of the CEP-1/p53-mediated apoptotic pathway. Data represent the mean fold change from three independent biological replicates. Statistical significance was determined using one-way ANOVA (* $p < 0.05$; ** $p < 0.01$).
