# Supplementary material for: Depletion of tryptophanyl-tRNA synthetase and tryptophan accumulation triggers p53-dependent apoptosis
Source: Cell Death Discov. 2025 Dec 5;12:34. doi: 10.1038/s41420-025-02887-x (PMC12824228; doi:10.1038/s41420-025-02887-x)
Supplement: Supplementary file 4 — Supplementary Fig. S4. [file 41420_2025_2887_MOESM4_ESM.pdf]

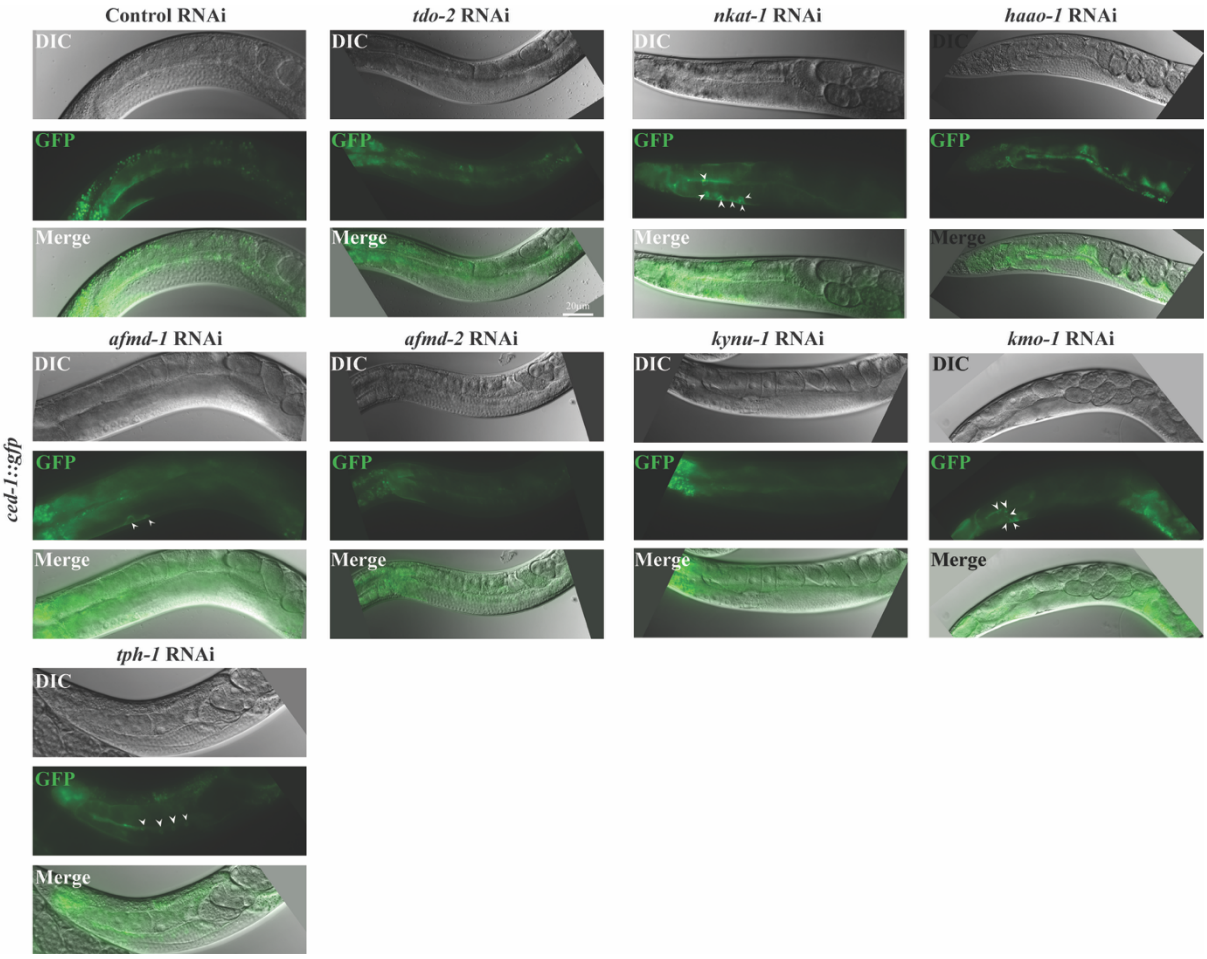

Supplementary Figure S4. Quantification of germ cell apoptosis upon knockdown of genes in the tryptophan degradation pathway. Apoptotic germ cells were quantified following RNAi-mediated knockdown of genes encoding enzymes in the tryptophan degradation pathway, including *tdo-2*, *nkat-1*, *haao-1*, *afmd-1*, *afmd-2*, *kynu-1*, *kmo-1*, and *tph-1*. The number of apoptotic cells was determined in the germline of *C. elegans* strain MD701 [*bcIs39 V (lim-7p::ced-1::gfp + lin-15(+))*], where apoptotic cells are visualized as CED-1::GFP–positive cells (arrowheads). Among the genes tested, only depletion of *nkat-1*, *kmo-1*, and *tph-1* resulted in a significant increase in germ cell apoptosis compared with control RNAi-treated worms.
