# Supplementary material for: Depletion of tryptophanyl-tRNA synthetase and tryptophan accumulation triggers p53-dependent apoptosis
Source: Cell Death Discov. 2025 Dec 5;12:34. doi: 10.1038/s41420-025-02887-x (PMC12824228; doi:10.1038/s41420-025-02887-x)
Supplement: Supplementary file 5 — Supplementary Fig. S5. [file 41420_2025_2887_MOESM5_ESM.pdf]

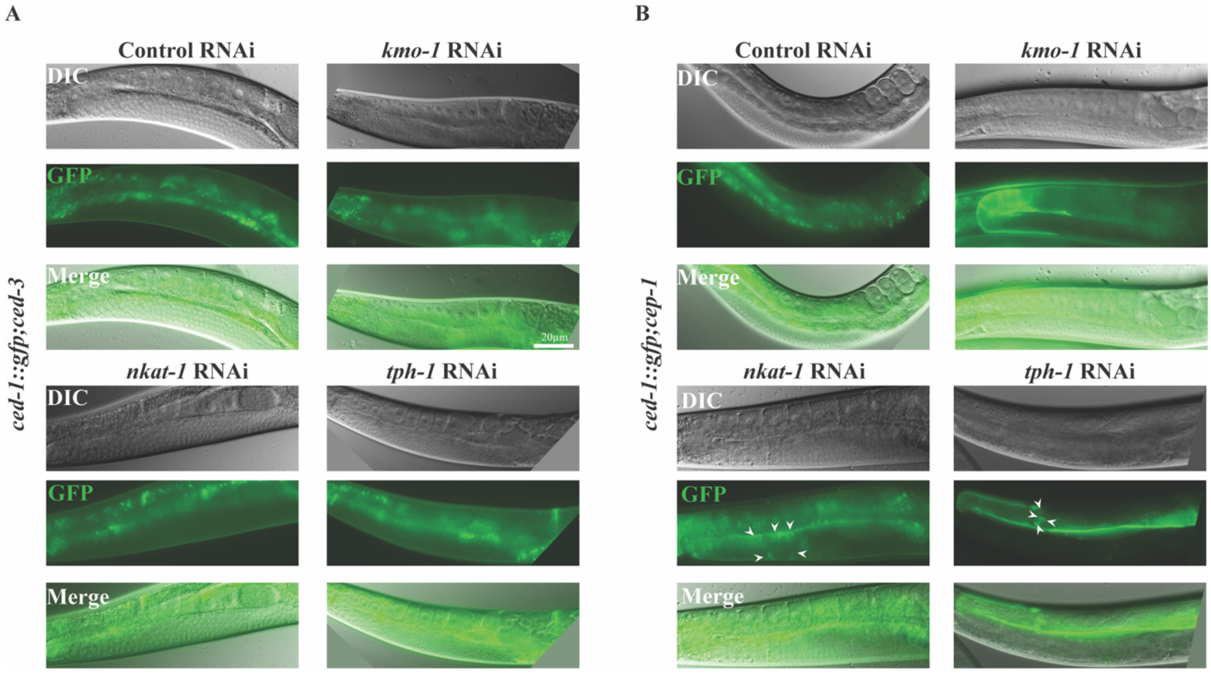

Supplementary Figure S5. Caspase and CEP-1/p53 dependency of apoptosis induced by depletion of tryptophan degradation enzymes. A. Quantification of apoptotic germ cells in *ced-1::gfp;ced-3(n2452)* caspase-null mutants following RNAi-mediated knockdown of *kmo-1*, *nkat-1*, and *tph-1*. Loss of *ced-3* function abolished the apoptosis observed in wild-type worms, demonstrating that cell death induced by disruption of tryptophan catabolism requires caspase activity. **B.** Quantification of apoptotic germ cells in *ced-1::gfp;cep-1(lg12501)* null mutants following RNAi knockdown of *kmo-1*, *nkat-1*, and *tph-1*. The apoptotic phenotype induced by *kmo-1* depletion was abolished in *cep-1/p53* mutants, indicating *cep-1*-dependent apoptosis. In contrast, *nkat-1* and *tph-1* knockdowns continued to induce germ cell apoptosis in the *cep-1/p53* null background, suggesting that their effects on apoptosis are independent of CEP-1/p53 function.
