# Supplementary material for: Depletion of tryptophanyl-tRNA synthetase and tryptophan accumulation triggers p53-dependent apoptosis
Source: Cell Death Discov. 2025 Dec 5;12:34. doi: 10.1038/s41420-025-02887-x (PMC12824228; doi:10.1038/s41420-025-02887-x)
Supplement: Supplementary file 6 — Supplementary Fig. S6. [file 41420_2025_2887_MOESM6_ESM.pdf]

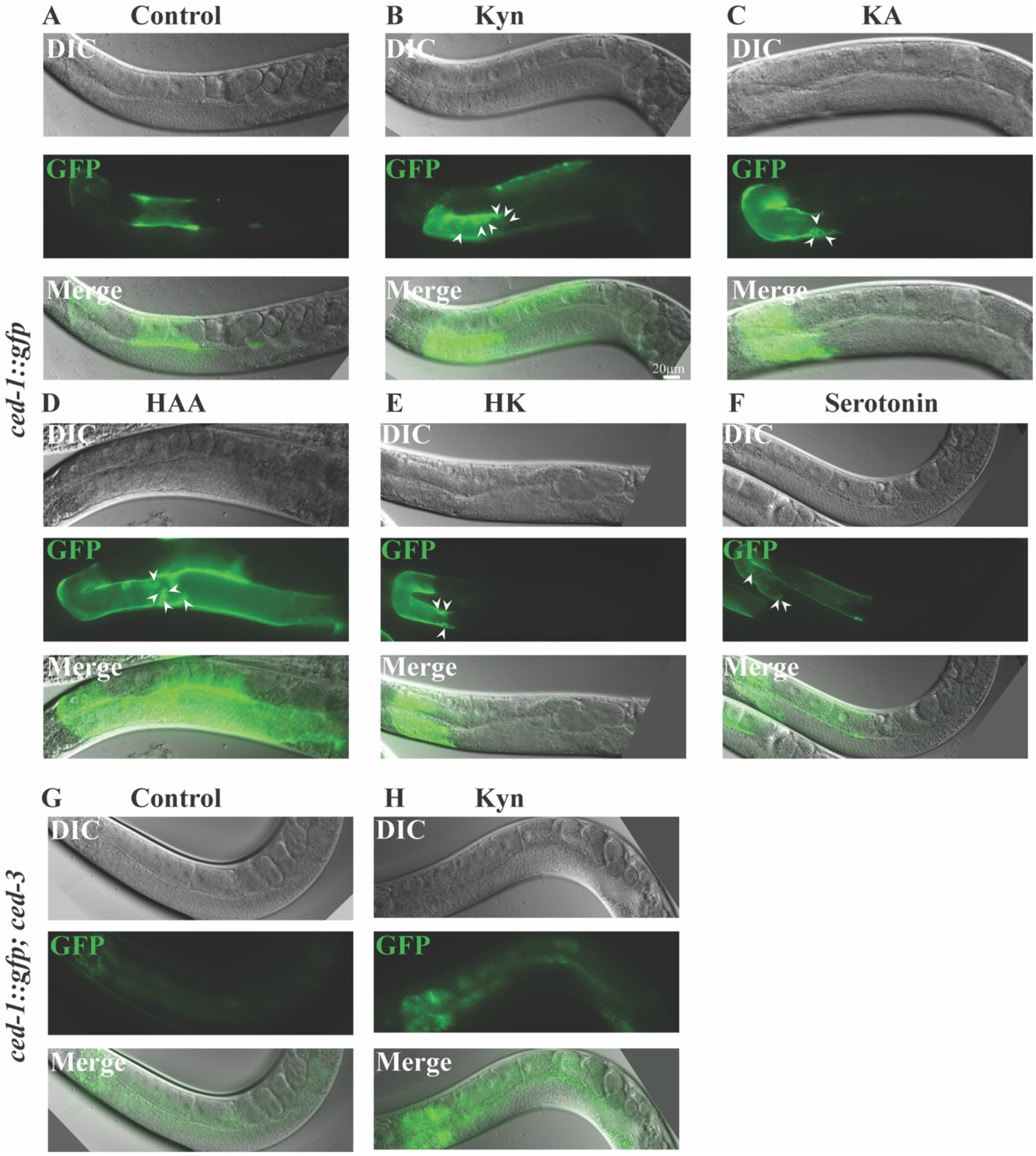

Supplementary Figure S6. Effect of tryptophan degradation pathway metabolites on germ cell apoptosis. Representative fluorescence images of *C. elegans* strain *ced-1::gfp* showing apoptotic germ cells in **A.** control worms and worms supplemented with **B.** kynurenine (Kyn), **C.** kynurenic acid (KA), **D.** hydroxyanthranilic acid (HAA), **E.** hydroxykynurenine (HK), and **F.** serotonin. L4-staged worms were exposed to the indicated metabolites until adulthood, and apoptotic cells were visualized as CED-1::GFP–positive germ cells (arrowheads). Among the metabolites tested, only kynurenine supplementation induced a pronounced increase in germ cell apoptosis (**B**). **H.** This kynurenine-induced apoptotic phenotype was abolished in *ced-3* caspase-null mutants, confirming that the effect is caspase dependent.
