# Supplementary material for: Depletion of tryptophanyl-tRNA synthetase and tryptophan accumulation triggers p53-dependent apoptosis
Source: Cell Death Discov. 2025 Dec 5;12:34. doi: 10.1038/s41420-025-02887-x (PMC12824228; doi:10.1038/s41420-025-02887-x)
Supplement: Supplementary file 7 — Supplementary Table S1. [file 41420_2025_2887_MOESM7_ESM.pdf]

Supplementary Table S1. List of primers used for amplification of coding sequences of *tph-1* and *afmd-2* from *C. elegans* genomic DNA (gDNA).

Gene name	Primer Code	Primer Sequence
tph-1	EP677	5'- CCGGGCCCGACAATCGCATGGAGGATTT -3'
	EP678	5'- TGGCGGCCGCGCGAACGTATTGAGTGCAGA -3'
afmd-2	EP701	5'- ACCGGGCCCTCATCGATTGAACGGCA -3'
	EP702	5'- GTGGCGGCCGCCTTGAAATGCGAGGT -3'
