# Supplementary material for: Depletion of tryptophanyl-tRNA synthetase and tryptophan accumulation triggers p53-dependent apoptosis
Source: Cell Death Discov. 2025 Dec 5;12:34. doi: 10.1038/s41420-025-02887-x (PMC12824228; doi:10.1038/s41420-025-02887-x)
Supplement: Supplementary file 8 — Supplementary Table S2. [file 41420_2025_2887_MOESM8_ESM.pdf]

Supplementary Table S2. List of primers used for quantitative real-time PCR (qRT-PCR) analysis of *egl-1* and *γ-tubulin* expression.

Gene name	Primer Code	Primer Sequence
egl-1	EP 399	5'-CCTCA ACCTCTTCGGATCTT-3'
	EP 400	5'-TGCTGATCTCAGAGTCATCAA-3'
γ-tubulin	EP 403	5'-AAGATCTATTGTTCTACCAGGC-3'
	EP 404	5'-CTTGAACTTCTTGTCCTTGAC-3'
